# Assessment of Objective Ambulation in Lower Extremity Sarcoma Patients with a Continuous Activity Monitor: Rationale and Validation

**DOI:** 10.1155/2014/947082

**Published:** 2014-12-25

**Authors:** Kenneth R. Gundle, Stephanie E. Punt, Ernest U. Conrad III

**Affiliations:** Department of Orthopaedics & Sports Medicine, University of Washington Medical Center, 1959 Pacific Street NE, P. O. Box 356500, Seattle, WA 98195, USA

## Abstract

In addition to patient reported outcome measures, accelerometers may provide useful information on the outcome of sarcoma patients treated with limb salvage. The StepWatch (SW) Activity Monitor (SAM) is a two-dimensional accelerometer worn on the ankle that records an objective measure of walking performance. The purpose of this study was to validate the SW in a cross-sectional population of adult patients with lower extremity sarcoma treated with limb salvage. The main outcome was correlation of total steps with the Toronto Extremity Salvage Score (TESS). In a sample of 29 patients, a mean of 12 days of SW data was collected per patient (range 6–16), with 2767 average total steps (S.D. 1867; range 406–7437). There was a moderate positive correlation between total steps and TESS (*r* = 0.56,  *P* = 0.002). Patients with osseous tumors walked significantly less than those with soft tissue sarcoma (1882 versus 3715, *P* < 0.01). This study supports the validity of the SAM as an activity monitor for the objective assessment of real world physical function in sarcoma patients.

## 1. Introduction

Multimodal treatment of lower extremity sarcoma has achieved gains in mortality alongside a shift to limb salvage over the past several decades [1]. While continuing to assess oncologic outcomes, there is a growing focus on measuring health-related quality of life and function with the use of patient-reported outcomes (PRO) [2]. Assessing these patient-centered outcomes is important for conducting comparative effectiveness research [3].

One assessment of functional outcome through a PRO measure is the Toronto Extremity Salvage Score (TESS) [4]. The TESS is a valid and reliable PRO measure designed to assess physical function and impairment following treatment for sarcoma. As an adjunct or alternative to survey-based PRO, objective measurement of walking performance in daily life may be valuable to understand how patients function, evaluate treatment options, and better educate patients on anticipated outcomes.

The StepWatch (SW) Activity Monitor (Orthocare Innovations, Seattle, WA) is a two-dimensional accelerometer used to evaluate walking patterns and intensity of activity. It has a high level of reliability in users with and without motor impairment [5] and is more accurate than standard pedometers [6]. Unlike gait lab analysis or functional tests performed in clinic, accelerometers measure patient activity in their daily lives and environments. The SW has previously been used to determent the number of cycles faced annually by total joint replacements [7] and has increased understanding of polyethylene wear rates [8]. A recent study reported on the validity of the SW accelerometer in adolescents who underwent limb salvage [1].

The purpose of this study was to validate the StepWatch Activity Monitor in a population of adult lower extremity sarcoma patients. The primary outcome was the correlation between total steps and the TESS. Secondary outcomes included total daily step count in osseous versus soft tissue tumors.

## 2. Methods

In a cross-sectional population of patients with lower extremity sarcoma treated with limb salvage at a single institution between 2010 and 2012, patients were recruited to wear the StepWatch as part of a prospective cohort. Patients provided signed informed consent on an institution-approved study protocol. All patients with lower extremity sarcoma were eligible. For the purpose of this validation study, patients were eligible whether receiving treatment for primary or recurrent disease. Patients were excluded if they chose not to participate, or if no devices were available at the time of their clinical visit. Clinical events such as readmission or complications did not exclude enrollment or participation.

The StepWatch device was calibrated per manufacturer recommendation using a manual count of 100 strides. Participants were instructed to attempt wearing the monitor for at least seven consecutive days during all waking hours except when swimming or bathing. Participants also completed the lower extremity version of the TESS. Demographic and oncologic characteristics were abstracted. Participants with inadequate monitoring, defined as wearing the monitor upside down, incorrect positioning, or not wearing the monitor for more than three hours during a day, were eliminated from analysis. Patients without complete TESS results were also excluded.

Descriptive statistics including mean and standard deviation were calculated for all variables. Student's *t*-test was used to compare continuous variables, and Fisher's exact test was used to compare proportions. Pearson correlation was used to test for correlation between total steps and TESS. All analyses were performed with Stata 11.0 (College Station, TX).

## 3. Results

Twenty-nine paired evaluations met inclusion criteria for the study. Patient demographics are detailed in Table 1. Fifteen (52%) were from patients with osseous sarcoma, and fourteen had soft tissue sarcomas. All underwent limb salvage surgery. Average time from surgery to SW use was 519 days (range 12–1932 days). A mean of 12 days of data collection provided SW data (range 6–16), with 2767 average total steps (S.D. 1867; range 406–7437).

The group with osseous tumors was similar to the group with soft tissue tumors (see Table 2), in terms of gender (*P* = 0.81), time from surgery (*P* = 0.25), and number of days in which the SAM was worn (*P* = 0.42), but the osseous group was younger (average age 45 versus 60, *P* = 0.03). The osseous tumor group took significantly less total steps than the soft tissue group (1882 versus 3715, *P* < 0.01). TESS was also lower in the osseous tumor group (68 versus 81, *P* = 0.02).

There was a moderate positive correlation between total steps and TESS (*r* = 0.56, *P* = 0.002), with higher total step counts being associated with higher scores on the TESS.

## 4. Conclusions

In a cross-sectional population of lower extremity sarcoma patients treated with limb salvage at a single institution, total steps as measured by the StepWatch Activity Monitor (SAM) were positively correlated with the TESS. Patients with osseous tumors were significantly less active than patients with soft tissue sarcomas and reported lower TESS results.

Patients who reported higher lower extremity function on the TESS tended to take more daily steps. This moderate positive correlation with the TESS, a widely used PRO measure of physical function in this population, supports the validity of the SAM. The lower total step counts and TESS results in osseous tumors, when compared with soft tissue sarcomas, also provide face validity as patients with osseous tumors generally undergo a more extensive endoprosthetic reconstruction. Likewise, these results provide another means of validation of the TESS as a PRO measure of physical function in patients with sarcoma, through a direct comparison to real world physical activity.

These findings are consistent with a recent study of the SAM in adolescents with sarcoma, which demonstrated lower total steps compared to age-matched controls and modest correlation with PRO measures [1]. While the present study lacked a control group, Silva et al. reported an average of 5,219 cycles, or over 10,000 steps per day, using the SAM in a study of 33 patients with well-functioning total hip arthroplasties [7]. Those patients, on average, achieved the widely cited public health goal of 10,000 steps a day after hip replacement [9]. In contrast, the current study shows far fewer daily steps, particularly in patients after limb salvage for osseous tumors. In addition to implications for advising patients regarding the expected outcome after surgery, such data may aid in evaluating real world wear rates and longevity in endoprostheses [8].

This cross-sectional validation study has limitations. Patient groups were similar regarding gender and the time from surgery, but osseous tumor patients were younger than those with soft tissue sarcoma. If anything, this might have been expected to show increased activity levels in the bone sarcoma group, which was not observed. While a strength of our design was that patients were not excluded based on perioperative complications, performance status, or whether receiving treatment for primary or recurrent disease, the current sample of 29 patients was not powered for subgroup analysis. Future studies would be able to assess time-dependent effects of treatment, complications, and recovery on activity levels.

There is possible selection bias, as patients elected to wear the activity monitor and we did not track outcomes on those who declined. Also, at our institution more than 120 new patients with soft tissue sarcoma present each year, indicating that the study population is only a subset of those undergoing treatment during the enrollment period. Those who wore the activity monitors may differ from the patients who were not included. While this reflects in part the limited number of activity monitors available, in subsequent studies it would be useful to ask and report on why potential participants choose to decline.

Assessing sarcoma treatment outcomes through PRO measures is critical to assess patient experiences of their disease and treatment [2]. Despite widespread use and enthusiasm, there remain critiques of survey-based outcomes. Some authors have noted residual or inherent subjectivity in survey-based outcomes, as well as epistemological questions as to whether we can capture an individual's quality of life or implicitly define “good” quality of life or physical function based on a questionnaire [10]. Real world activity monitoring addresses some critiques of PRO measures, but incorporation of such devices into clinical research adds significant cost and logistical challenges. Furthermore, patients with sarcoma have significant heterogeneity in their disease, adjuvant treatments, and invasiveness of surgical treatment that likely impact their activity. Concurrent use of PRO measures and activity monitors, when feasible, may inform on the relative strengths and weaknesses of each approach to describe outcomes that matter most to patients and their physicians.

Activity monitors give a window into how patients function in their daily lives, away from the clinic or gait labs. The information gained may facilitate the informed counseling of patients before surgery and during rehabilitation and in assessing treatment strategies. A growing body of literature supports the validity of these instruments, including their use in the US National Health and Nutrition Examination Survey [11]. This study supports the validity of one such activity monitor, the SAM, for investigating outcomes in adults with lower extremity sarcoma.

## Figures and Tables

**Table 1:** Patient characteristics.

Total (*n*)	29	
Female (*n*, proportion)	18 (62)	
Age (mean, range)	52 (22–76)	
Time from surgery, in days		
(mean, range)	519 (12 to 1932)	
Type		
Bone	16	55
Soft tissue	13	45

SAM data	Mean (SD)	Range

Days of use	12 (3)	6 to 16
Total daily steps	2767 (1867)	406 to 7437

**Table 2:** Osseous versus soft tissue tumors.

	Bone	Soft tissue	*P* value
Age (y)	45	60	0.03
Female (proportion)	0.6	0.64	0.81
Time from surgery (days)	645	395	0.25
SAM wear (days)	12	13	0.42
Total steps (mean, SD)	1882 (260)	3715 (570)	<0.01
TESS	68	81	0.02
